# Surveying the Professional Experience of Special Educational Needs Provision in England

**DOI:** 10.1111/cch.70227

**Published:** 2025-12-26

**Authors:** William Farr, Isaac Winterburn, Jacob Matthews, Jennifer Saxton, Tamsin Ford, the HOPE study

**Affiliations:** ^1^ Faculty of Education University of Cambridge Cambridge UK; ^2^ Department of Psychiatry University of Cambridge Cambridge UK

**Keywords:** experience, professionals, provision, SEND, survey

## Abstract

**Introduction:**

Support for children with Special Educational Needs and Disability (SEND) is in a state of flux. To investigate from the professional side, we compared experiences around identification of SEND and related provision for professionals working with young people and families in England.

**Methods:**

This was an online self‐reported cross‐sectional survey with education, health, social care, third‐sector and local authority professionals. The survey was co‐produced with service‐users and professionals essential to the design and implementation of SEND services. Here we report on professionals' opinions about the state of services offered and the reasons behind this.

**Results:**

Professionals reported polarised views surrounding interagency communication and quality, with many doubting the ability of services to adequately provide support for SEND. Professionals reported high confidence in their own abilities to identify SEND (86% agreed), design and provide services (58% agreed), communicate with parents, signpost to services and receive adequate training, but were less confident about others' abilities (62%) to communicate effectively. Professionals report insufficient time (74%) and poor resourcing to enable good design of plans and service quality (80%).

**Conclusion:**

Rising national and international demand for SEND services, alongside rising costs, has caused service delay and disrepair as key aspects of the educational ecosystem, e.g., communication and service development, have been whittled away. Systemic change is needed to reflect growing SEN school numbers, requiring fully integrated care for children and families.

## Summary

- Communication effectiveness between agencies is insufficient, whilst professionals are confident in their own ability.
- Professionals are confident in identifying SEND, signposting, designing and providing services
- Support is seen as insufficient for children and young people.
- More time is needed to design and deliver quality provision.

## 1 Introduction

Support for children in schools with Special Educational Needs and Disability (SEND) has been a consistently sensitive and contentious aspect of national and global educational debate over the last 30 years (Gordon‐Gould and Hornby 2023). Underpinning the debate are the principles of normalisation that led to inclusion alongside the increased drive to address needs wholly within mainstream settings, inadvertently closing the doors on many special needs settings (Gordon‐Gould and Hornby 2023; Warnock 1978; Warnock and Norwich 2010; Wolfensberger et al. 1972). The resulting current situation from the Code of Practice (2014) and the SEND Improvement Plan (2023) has led to an even greater push towards mainstream provision at the expense of provision for complex needs.

There is an acknowledged paucity in international literature on SEND provision, gaps persist between policy and practice, as well as how teachers remain core to inclusive provision, and opinions and attitudes amongst education professionals remain varied, especially amongst senior leaders (Banerjee et al. 2014; Hay et al. 2025). One aspect that persists across all regions, though, is that SEN and childcare are consistently under pressure, and when children struggle either with schooling or major disability it remains potluck as to the type of received care, even in the face of the Salamanca statement (Farr et al. 2026).

Approximately 1.6 million children in England with SEND were identified in 2024, with a 12% increase in the number of new Education Health Care Plans (EHCPs) issued from 2023—a drop from 26% in 2022–2023 (DfE 2024). Nearly 1‐in‐20 (4.7%) of all school children have EHCPs (DfE 2024), which defines the highest and legally protected level of need (Wolfe and Glenister 2024). In addition, 13.6% of all children in schools receive a lower tier ‘SEN support’, where there is no legal obligation to track, record and plan for provision, other than the mandate established by teachers themselves (Ofsted 2024). Yet, the ecosystem surrounding children, young people and families with SEND is in apparent disarray (Crane et al. 2016).

Identification of children with SEND usually begins with parents querying issues alongside teachers, SENCOs and/or primary health staff such as General Practitioners (Abrahamson et al. 2020, 2021). This leads to a referral and an assessment in either Paediatric or Mental Health Services. Meanwhile provision refers to the pragmatic actions that will take place to meet a child's needs, which can occur in one of two ways. Support may be offered within the boundaries of school activity, such as in‐school interventions or tiered support, or by responding to needs that are met by other services either outside school premises (such as sensory needs, physical therapy or autism support services) or on school premises (such as speech and language provision or educational psychology).

Professionals span health, education and social care, but also the third sector and charities, with recently an increasing number of private providers (Abrahamson et al. 2021; Farr et al. 2022).

The roles of practitioners from all these sectors can be highly specialised, such as Speech and Language Therapists, or more generalised like Educational Psychologists. Some children with medical or mental health conditions require input from Paediatricians or Psychiatrists or Allied Health Professionals such as Physiotherapists. However, the role and responsibilities of the Special Educational Needs Co‐ordinator (SENCO), which focuses on educational needs and provision and also extends to family support, have become increasingly pivotal in schools. The SENCO role, more than any other at the heart of support for education, reflects the state of provision—‘confused and contested’ (Curran 2019). Barriers and details around multi‐agency are reported by one of the authors elsewhere (Male et al. 2023, Male and Farr 2020, Parr et al. 2024) shows that where teams co‐locate, use a multidisciplinary team, use a clear pathway to care and are clear about paperwork, definitions and points of access this improves care and access to help for SEND. Further, where clinical teams are moving towards broader Neurodevelopmental pathways, rather than single issue pathways (e.g., ADHD, Autism), then care and clarity improves.

The SEN and/or Disabilities and Alternative Provision (AP) Green paper (Department for Education 2022) shows that the SEND system faces three key challenges: poor outcomes for children and young people with SEND who may need alternative provision, overall costs and low parent and provider confidence in the system. To tackle the problem, three programmes of work were planned: the Safety Valve Intervention, The Delivering Better Value (DBV) in SEND and the Education and Skills Funding Agency programme. Working with local authorities (LA) DBV aims to appropriately manage: (i) ‘demand for EHCPs, including assessment processes that are fit for purpose and (ii) use of appropriate and cost‐effective provision, which includes ensuring mainstream schools are equipped and encouraged to meet needs where possible, whilst maintaining high standards for all pupils’ (Department for Education 2022, p10). Within this context we aimed to construct and survey professionals involved in the SEND process. What is missing from the literature is known shortfalls in professional understanding and the breakdown of areas of need in accordance with the profession. Prior work either focuses on parents alone or problems from an internal (e.g., within an NHS trust) rather than an interagency perspective, particularly where professionals are asked to judge their own and others' knowledge and capacity (e.g., Crane et al. 2016; Farr et al. 2022).

## 2 Rationale

The survey was constructed to discover the experience of professionals within the process of *identification* for children with SEND. This would highlight professionals' views and opinions on the process. Our survey tool was co‐produced to ensure it was relevant to service users and those designing and implementing SEND services. This survey was part of the HOPE Study (**H**ealth **O**utcomes of young **P**eople throughout **E**ducation), a collaboration between University of Cambridge and University College London and funded by the National Institute for Health Research Programme for Applied Health Research (NIHR). The HOPE Study investigated the impact of adjustments for special educational needs (SEN) on children's health using linked education and hospital data for all children in England (the https://www.ucl.ac.uk/child‐health/echild).

We co‐produced three online self‐report cross‐sectional surveys with three public and patient involvement (PPI) groups comprising (1) young people with SEND, (2) parents/carers and (3) professionals to ensure survey questions were relevant to the issues currently being experienced by service users and those essential to the design and implementation of SEND services. We invited children and young people with SEND (CYP), parents and carers of children with SEND and SEND professionals working closely with young people across education, health and social care (SP) to complete our online survey. A separate paper contrasts responses from comparable questions across all three stakeholder groups, whilst another focuses on questions unique to CYP and parents/carers (Survey Paper 1 and 2 in separate submission). Here we report on the professionals' experience.

## 3 Methods

### 3.1 Ethics

Ethical approval was granted by the Cambridge Psychology Research Ethics Committee for this study (PRE.2021.058). Participant information sheets, consent forms, interview schedules for focus groups and the observer proforma are all attached as appendices to this article.

### 3.2 Study Design

A cross‐sectional survey was constructed and a convenience sample of SEND professionals in England was participants. Convenience sampling is a good method to employ when the population is unknown, large and available but not easy to reach, and is so suitable for an online distributed survey. However, there are drawbacks, such as it is not always fully representative and so can be subjective in selection, but overall it is seen as useful when time, resources and workforce are limited (Etikan et al. 2016).

Sample size was not calculated as this was a convenience sample seeking as many professionals as possible from as wide a reach as possible across geographical areas in England. The sample size was out of an unknown total population of professionals who work with children with special needs, but a minimum calculated sample would be at least 70 respondents (Teare et al. 2014), which this study exceeded.

### 3.3 Survey Co‐Production and Development

Each survey was co‐designed via an iterative and piloting process with advisory groups that were associated with the HOPE study (CYP, parents/carers, SEND Professionals), and who submitted approximately 300 potential questions around identification, assessment and provision of SEND. Representation was taken from multiple minority and SES groups, as well as from all professions discussed in the survey. The survey was piloted through RedCap, which is a secure web application for making and administering online surveys and databases, and advisory groups gave iterative feedback on 5 versions of survey content before the survey went live.

Co‐production led to the development of survey schedules through a lengthy iterative process; selection of topics and questions was through an expert advisory group and outputs with scoping evidence collated together with opinions made up of stakeholders, parents, family members and children with support; questions were pilot tested on live subject groups to check for ambiguity and clarity with an eye to criticality. Finally, the survey was run live.

### 3.4 Survey Items

The survey included questions about participant demographics (gender, region, ethnicity), professional role, experiences of working as a SEND professional at the SEND identification and provision stages. Response options varied by question, including yes/no, multiple and single checkboxes and Likert scales. Data S1 lists the questions used to compare SEND professionals' responses in the current paper. Questions were prioritised based on earlier scoping and systematic review as well as input from the research team and advisory group. Pilot testing was conducted over four iterations over a period of 1 month to check for content with a random sample of participants.

### 3.5 Eligibility Criteria for Survey Respondents

SEND professionals that have worked in or closely with education, health, LAs and other (e.g., third sector, private sector) settings and are involved in working with children who had SEND within the last 2 years were eligible to take part.

### 3.6 Recruitment of Survey Respondents

Online survey recruitment took place through social media and professional groups across England. Recruitment efforts prioritised responses from all areas of the country. Snowballing recruitment was used, and contacts across all 151 LAs in England responsible for SEND provision were identified from:

- Local Offer webpage admin/contact
- SEND Information, advice and support services (SENDIASS)
- Parent Carer forum/support group
- Children and young people's forum/support group
- Extracurricular club, group or activity

Participation was advertised across all HOPE study advisory group members and SEND contacts within England's universities. Other institutions were also invited to take part and distribute the survey: National Association for Special Educational Needs, Special Needs Jungle, The Association for Child and Adolescent Mental Health and ~24 000 schools through the Gov.uk notify service (https://www.notifications.service.gov.uk/). Surveys links appeared on the study webpage, and other social media spaces such as Twitter, Facebook, Instagram and WhatsApp.

### 3.7 Data Collection Procedure

Data was collected between 4th July and 10th October 2022, allowing for participation by retirees, staff moving or changing roles, including over the English summer holiday break whilst the survey was live. The survey was hosted on RedCap, accessed via a single hyperlink that asked participants to select the survey for which they were eligible. Individuals logged into RedCap and data was anonymised at source; no IP addresses or identifiable data—other than that given—was retained. Information sheets and online consent forms were provided whereafter signed consent participants could progress to complete the survey.

Following survey completion, debriefing information provided links to further information and support, thanked people for participation and encouraged survey link sharing to other interested parties. In addition, professionals and parents were encouraged to complete a second survey if they inhabited dual roles. Accessibility options were offered to participants, including enlarged text, text‐to‐speech function, easy‐read and the option to complete the survey over the phone.

### 3.8 Data Analysis

Data was exported from RedCap, de‐identified and cleaned with Microsoft Excel. Descriptive statistics (percentage and number) were disaggregated by professional role, as well as for SEND professionals. Survey data was not included if there were fewer than five responses as this could result in deanonymisation of respondents.

## 4 Results

### 4.1 Participants

Overall, 863 (75% of those approached) professionals completed the survey. There were 271 RedCap responses excluded because of incompletion or consent issues. A further 13 were removed because they were deemed to be accidental duplicate responses. This reduced 1147 responses down to the sample size of 863. Of these (see Data S3), most, 641 (74.3%) were education professionals, with 120 (13.9%) health professionals, 68 (7.9%) LA professionals and 34 (3.9%) ‘Other’ professionals working in the third sector or independent practice. Social care professionals were grouped under LA professionals. There were six social workers and six family support workers. Participants were predominantly of white ethnic background and female. There was engagement from all health regions of England, but greater representation from the Southeast and East of England. Data S2 provides participants' demographic information in further detail. Social care professionals were underrepresented in the survey and collapsed in the analysis phase into either local authority (68 or 7.8% of the total sample) or ‘other’ (34, 3.9%) professionals as there were so few. Education professionals were by far the most represented profession (641, 74%) perhaps due to response rate, as all local modes for distributing the questionnaire (local authority, children's services, universities, email, social media) were made.

### 4.2 Identification

**FIGURE 1:**
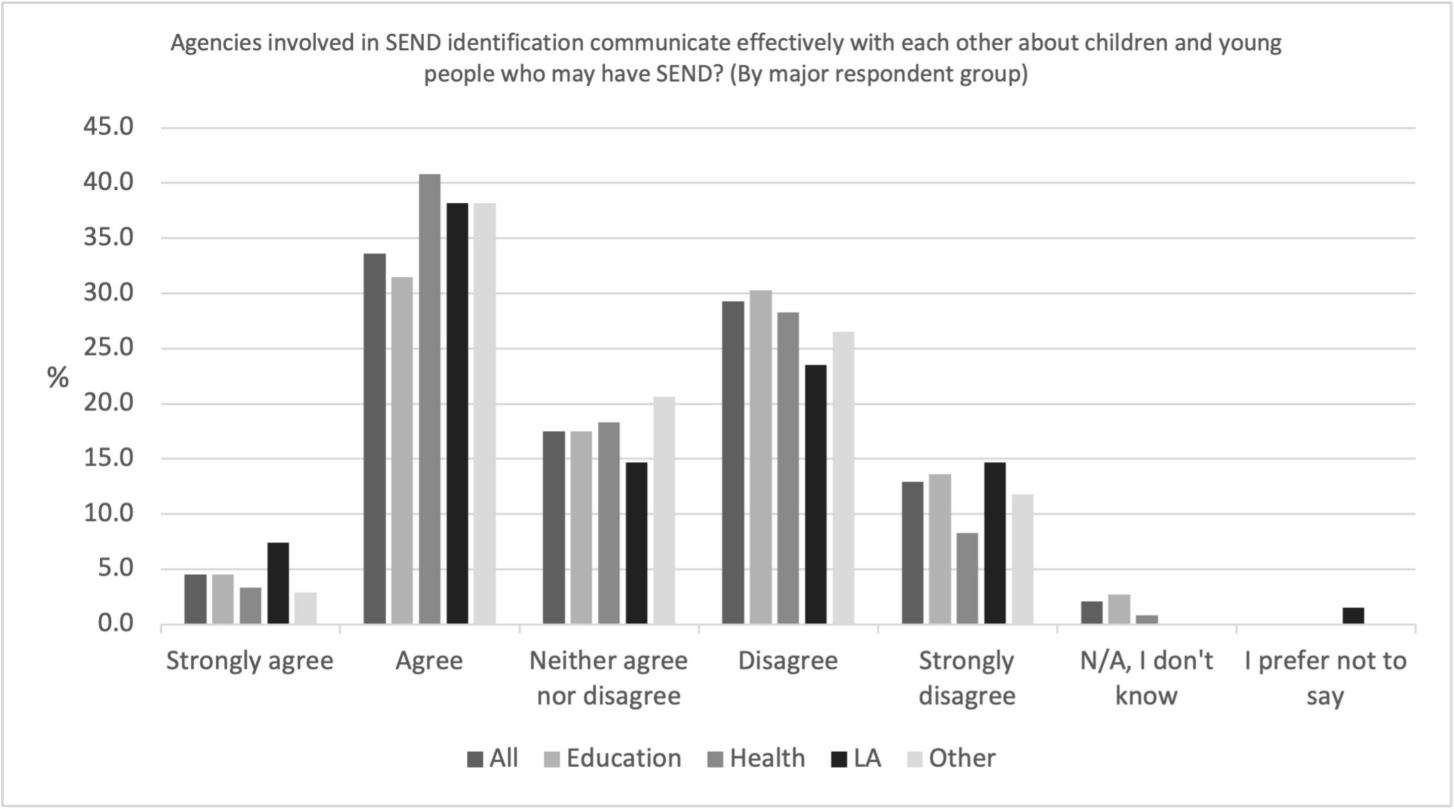
Professional communication effectiveness by occupation.

Figure 1 shows professional communication effectiveness by profession, and that approximately one‐third of SEND professionals (38%, *N* = 329) agreed or strongly agreed that in the LA/practice where they spent most of their time, agencies involved in SEND identification communicate effectively with each other about children and young people who may have SEND. Agreement with this statement was slightly higher amongst LA staff and health professionals (45.6%, *N* = 31 and 44.2%, *N* = 53 respectively), than Other (41.2%, *N* = 14) and Education professionals, (36.0%, *N* = 231). Figure 1 suggests approximately half of respondents do not agree that communication was effective, but the neutral category (neither agree nor disagree) suggest that between approximately 15%–20% of professionals, were unsure and non‐committal.

**FIGURE 2:**
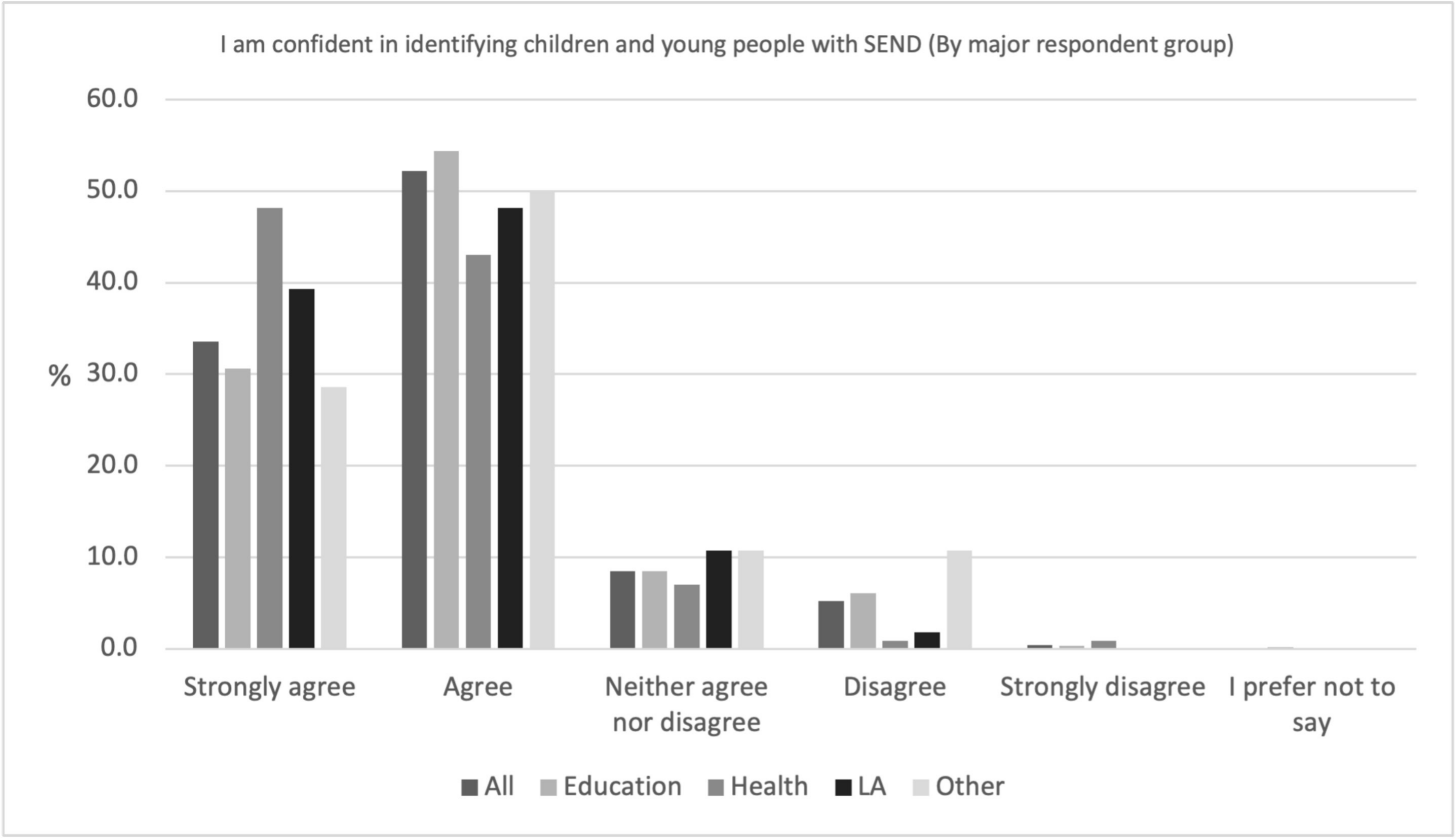
Professional confidence in the identification of SEND by occupation.

Figure 2 shows professional's response to the statement ‘I am confident in identifying children and young people with SEND’, and personal confidence in respect of this, of which 85.8% (*N* = 708) of all professionals agreed or strongly agreed. Health professionals were the most confident (91.2% /*N* = 104) compared to 87.5% (*N* = 49) of LA professionals, 85% (*N* = 533) of education professionals and 78.6% (*N* = 22) of Other professionals.

**FIGURE 3:**
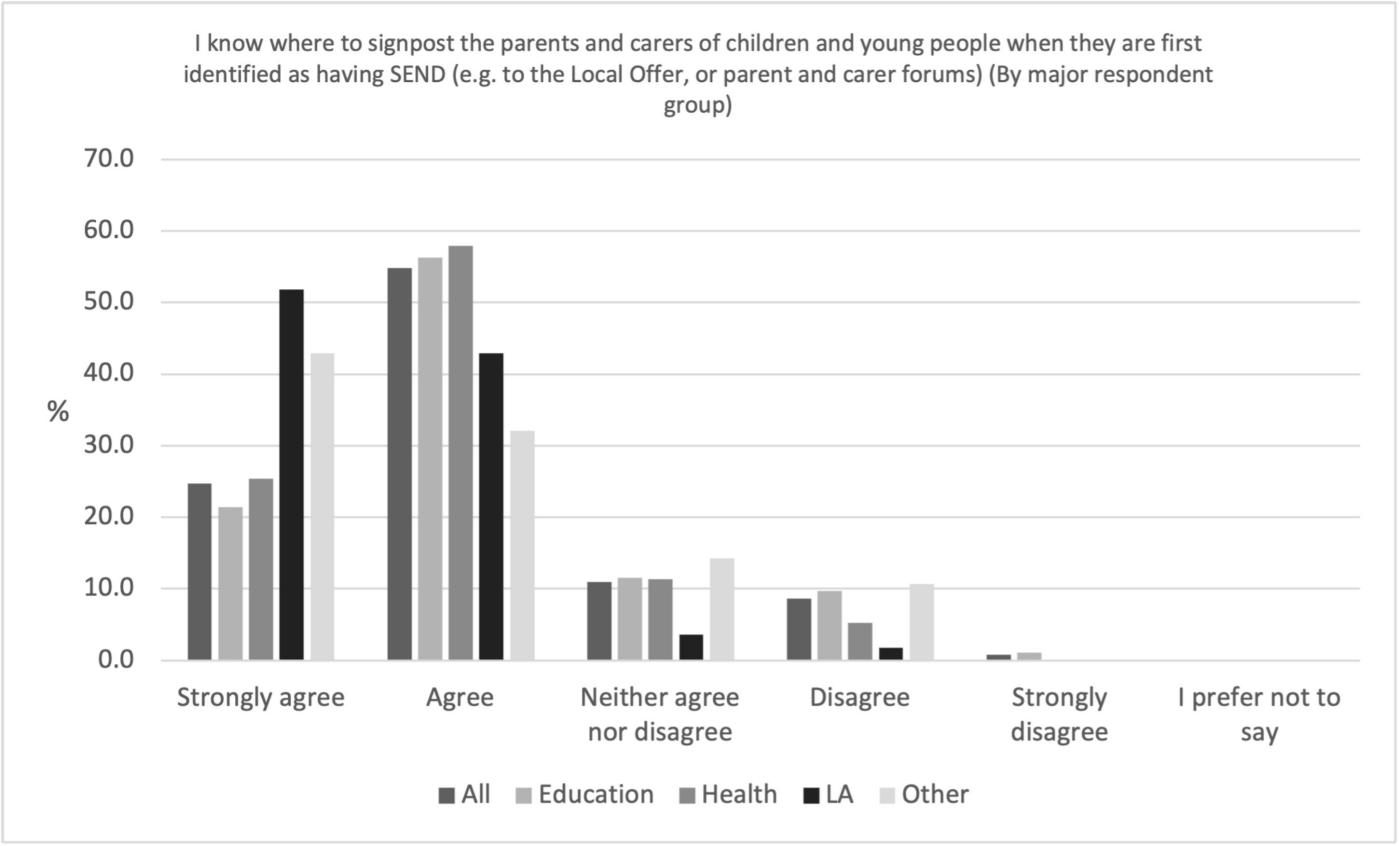
Professional knowledge of where to signpost by occupation.

As shown in Figure 3, 79.5% (*N* = 656) overwhelmingly professionals agreed or strongly agreed that they knew where to signpost the parents and carers of children and young people when they are first identified as having SEND. Whilst 94.6% (*N* = 83.3) of LA professionals agreed or strongly agreed to this statement, there was slightly less agreement from professionals in health (83.3%, *N* = 95), education (77.7%, *N* = 487) and Other settings (75%, *N* = 21). This variation between and within professionals is a continuing theme throughout these survey results.

### 4.3 Provision

**FIGURE 4:**
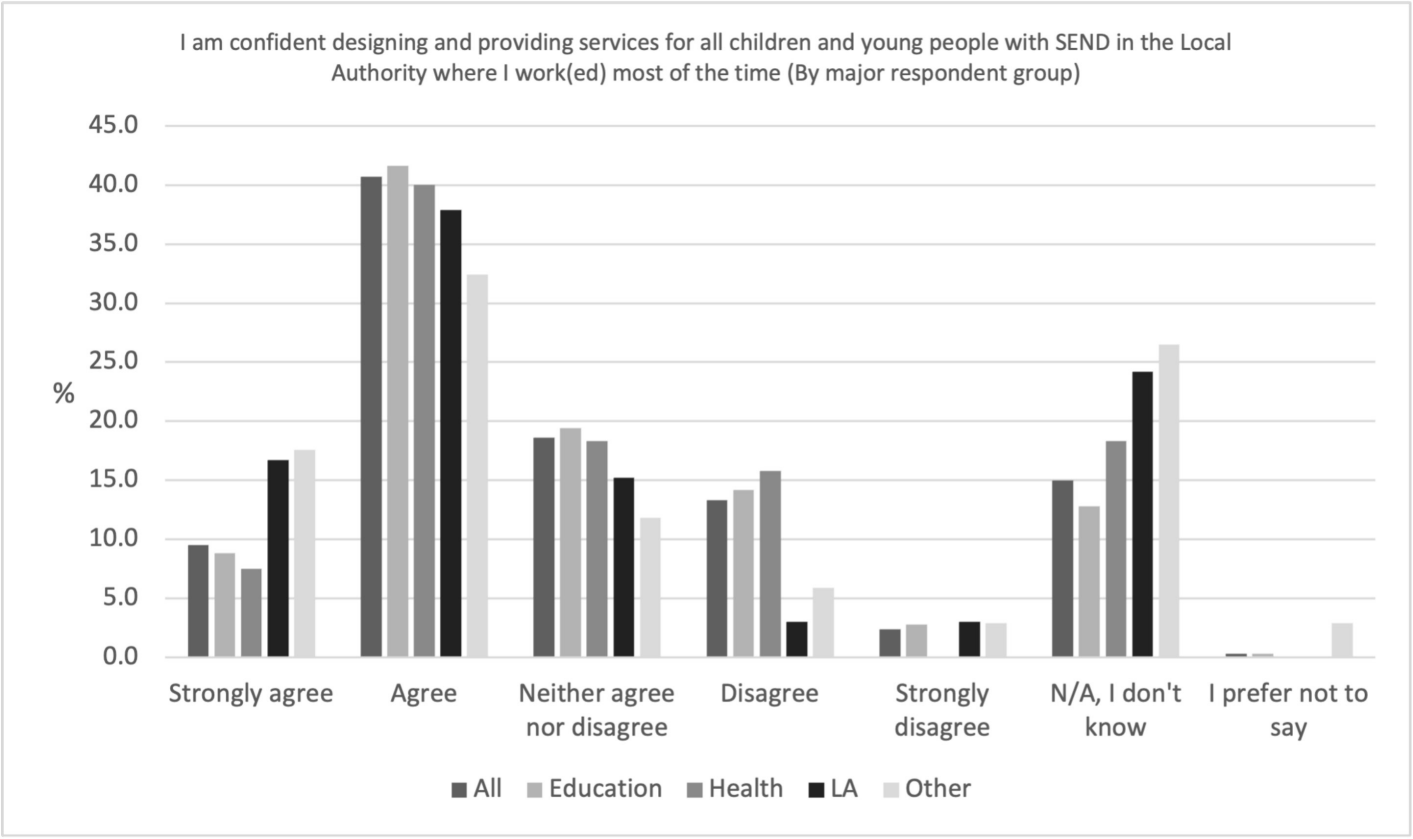
Professional confidence in designing and providing services by occupation.

In Figure 4, half of all professionals (50.3%, *N* = 432) agreed or strongly agreed to the statement ‘I am confident designing and providing services for all children and young people with SEND in the LA where I work (ed) most of the time’. So, professionals believe they have the skill to make good provision. Similar percentages of LA (54.5%, *N* = 36), education (52.0%, *N* = 322), Other (50%, *N* = 17) and health (47.5%, *N* = 57) professionals responded with ‘Agree’ or ‘Strongly agree’ (see Figure 4).

**FIGURE 5:**
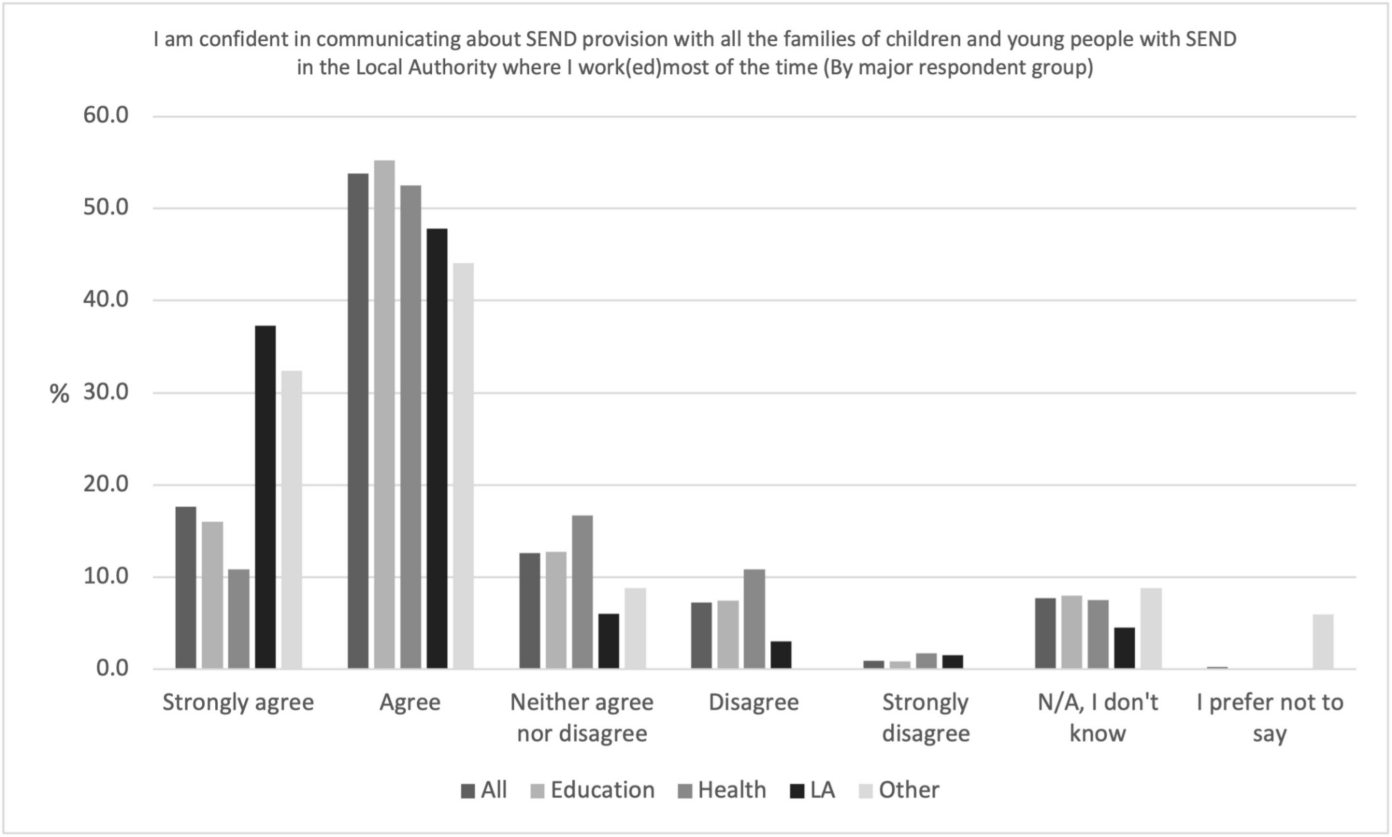
Professional confidence communicating about SEND provision by occupation.

Figure 5 shows how over 70% of all professionals (71.4%, *N* = 614) agreed or strongly agreed they felt confident in communicating about SEND provision with all the families of children and young people with SEND in the LA where they work (ed) most of the time. Similar percentages of Other (76.5%, *N* = 26) and education (71.2%, *N* = 455) professionals responded similarly. In addition, whilst 85.1% (*N* = 57) of LA professionals agreed or strongly agreed, only 63.3% (*N* = 76) of health professionals felt the same.

**FIGURE 6:**
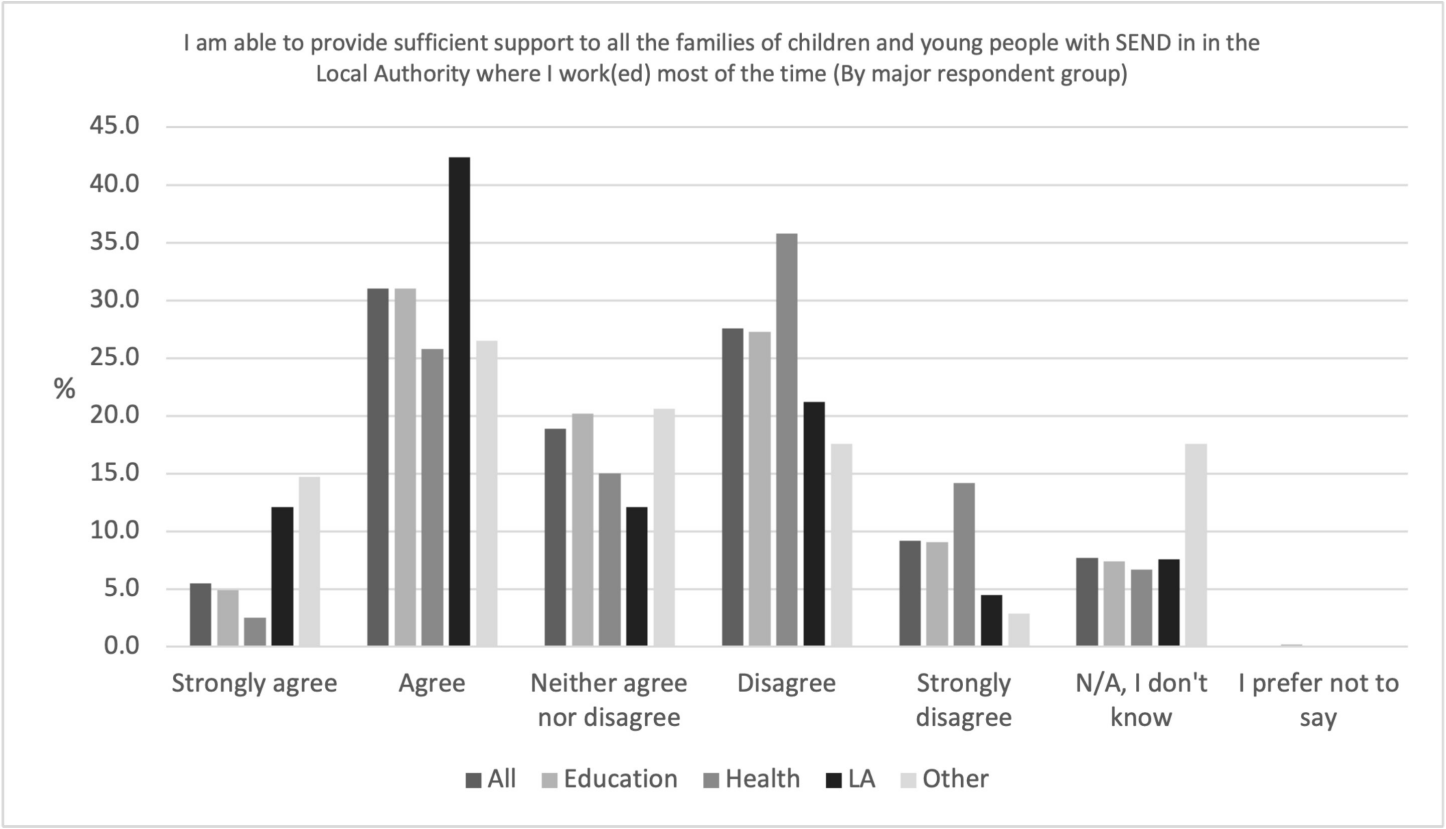
Professional ability to provide SEND support by occupation.

Professionals doubted their own ability to provide sufficient SEND support. Only 36.5% (*N* = 313) of all professionals agreed or strongly agreed to the statement ‘I am able to provide sufficient support to all the families of children and young people with SEND in the LA where I work (ed) most of the time’ (see Figure 6). Agreement was slightly higher for those working as LA (54.5%, *N* = 36) or Other (41.2%, *N* = 14) professionals compared to those in education or health: 35.9% (*N* = 229) and 28.3% (*N* = 34) respectively.

**FIGURE 7:**
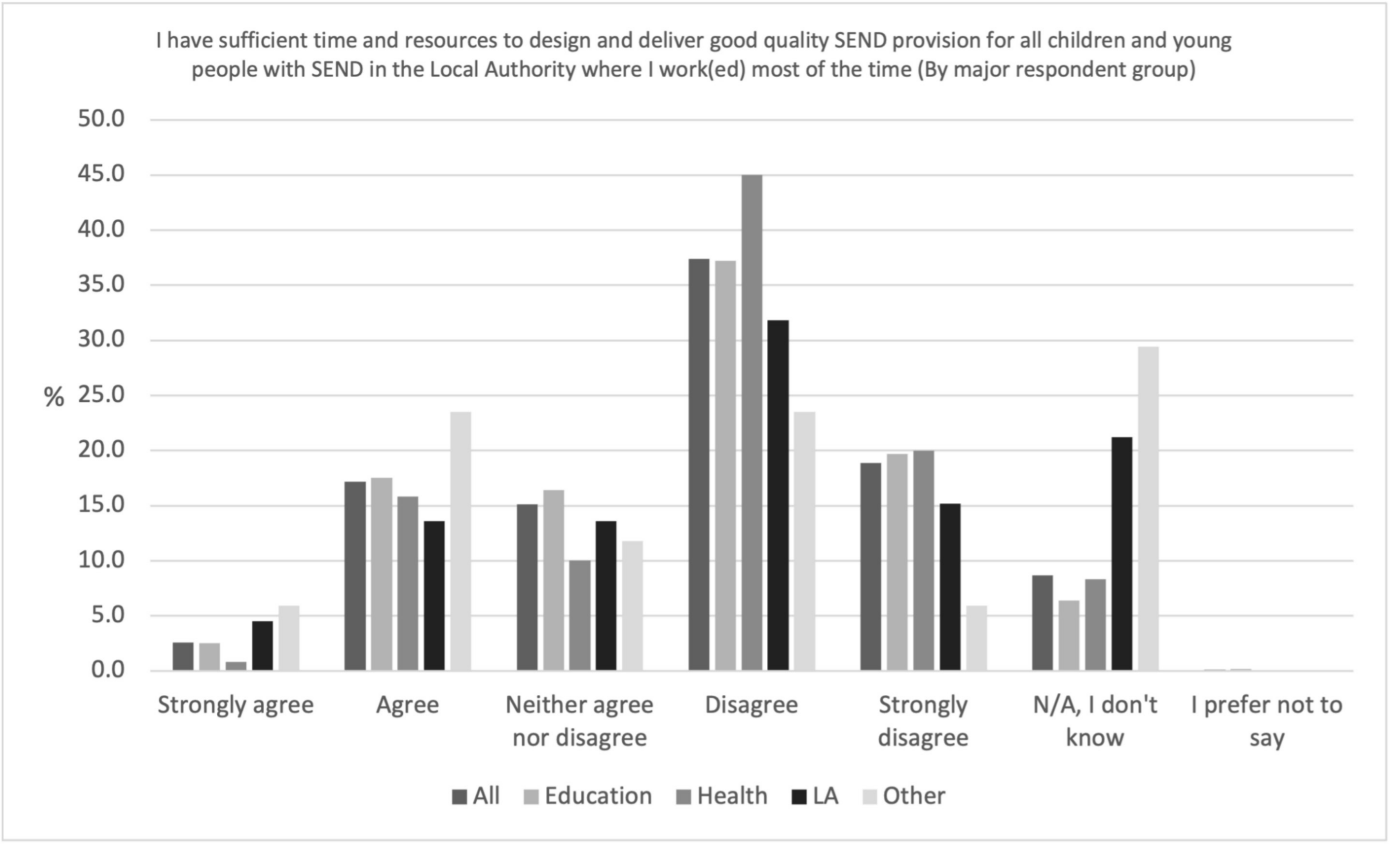
Professional time to design and deliver SEND provision by occupation.

Professionals believe they lack time and resources, as shown in Figure 7, only 19.8% (*N* = 170) of all professionals felt they had sufficient time and resources to design and deliver good quality SEND provision for all children and young people with SEND in the LA where they work(ed) most of the time. Agreement was highest amongst Other professionals and lowest for those working in health (16.7%, *N* = 20).

**FIGURE 8:**
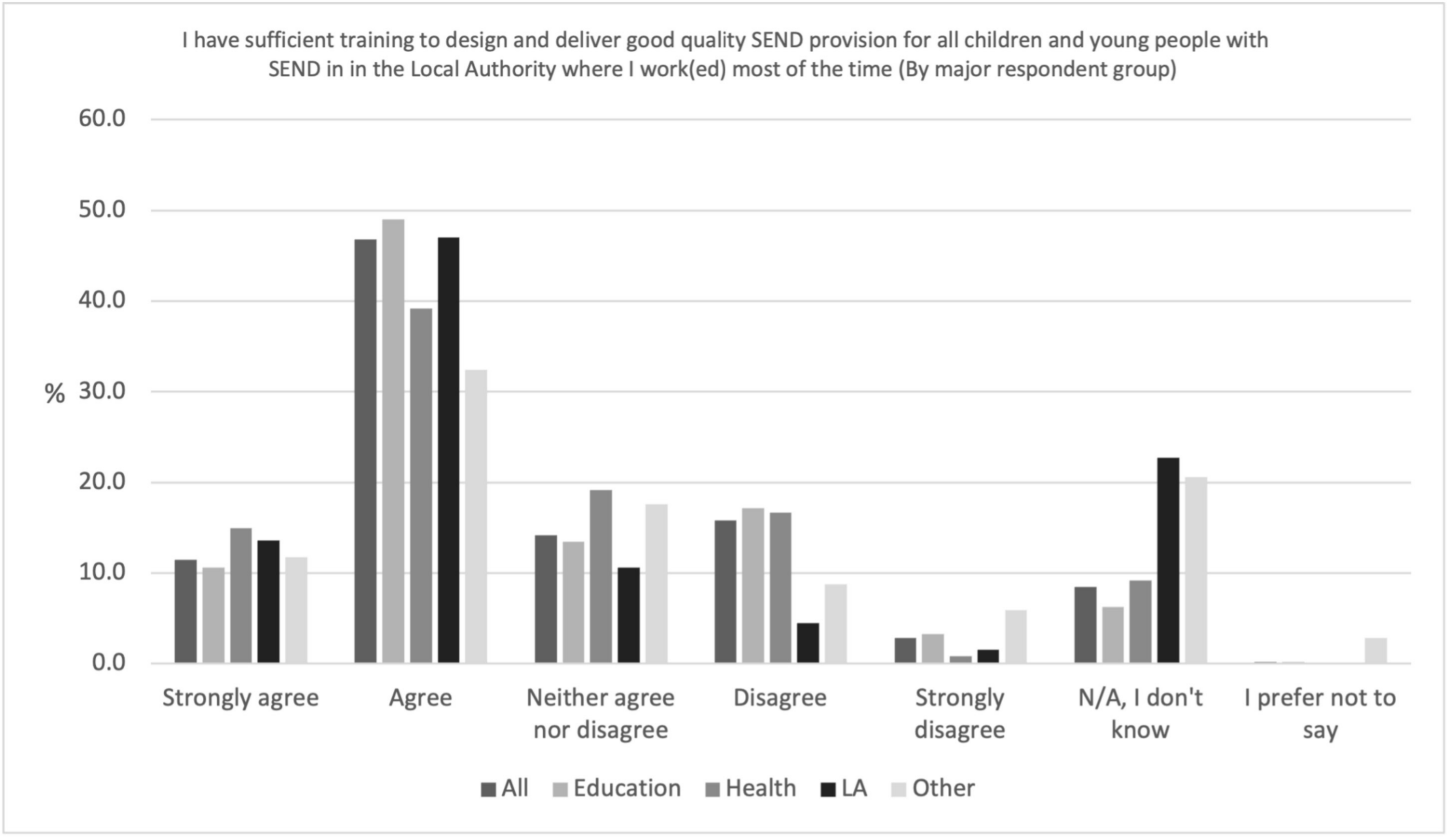
Professional training for the design and delivery of good provision by occupation.

Most professionals believed there was not enough training (see Figure 8) to design and deliver good quality SEND provision in the LA where they work(ed) most of the time. Over half (58.3%, *N* = 501) agreed or strongly agreed with the statement, similar to those within education (59.6%, *N* = 381) and health (54.2%, *N* = 65). However, 60.1% (*N* = 40) of LA professionals agreed or strongly agreed, whilst only 44.1% (*N* = 15) of Other professionals agreed.

### 4.4 Barriers to Services and Allocation of Resources

Data S3 provides full detail about what professionals perceived to be the main barriers to providing good quality SEND services for those who need them, as well as how they felt the allocation of resources for SEND was influenced in the LA/Practice where they work(ed) most of the time.

There was a consistency of responses across professionals from different sectors. Notably, < 0.5% selected that none of the barrier options applied in their LA. The most frequently selected barriers across the sample included ‘Lack of LA funding’ (501, 19.9%), ‘Length of waiting lists’ (463, 18.4%), ‘Insufficient access to SEND specialists’ (351, 13.9%), ‘Excessive caseload’ (251, 10.0%), ‘Lack of time’ (185, 7.3%), ‘Communication between different services/intersectoral working’ (168, 6.7%) and ‘Lack of training or expertise’ (131, 5.2%). The least selected barriers across the sample included ‘Burnout’ (30, 1.2%), ‘Communication with parents and carers’ (29, 1.2%), ‘Poor senior leadership’ (29, 1.2%), ‘Relationships with social care professionals’ (25, 1%), ‘Difficulties interpreting and applying the SEND Code of Practice’ (24, 1%), ‘Relationships with parents and carers’ (20, 0.8%) and ‘Relationships with education professionals’ (12, 0.5%).

There was a similar consistency with regards to what factors influenced the allocation of resources, with professionals most frequently selecting ‘By parent/carer ability to advocate for their children’ (239, 27.8%) and ‘By LA resources available’ (205, 23.8%). Surprisingly, 12.3% (106) of responses were for ‘I'm not sure’. The least commonly selected were ‘Based on first come‐first served’ (19, 2.2%) and ‘If a child/young person is in care or on the edge of care’ (9, 1.0%).

## 5 Discussion

This study has reported novel findings from a survey co‐produced with service‐users and SEND professionals about professionals' capacity to deliver high‐quality care to children with SEND in England. The main research question for this piece of work was to understand the experience of professionals when it came to identifying children with SEND. What was found was that views are very mixed amongst professionals with no clear and vision on identification. Within that, there is much gatekeeping over professional views, which are very guarded and positive towards their own work, and yet coming through clearly is a lack of resourcing to fully achieve all aims as required by law and the Code of Practice.

Across the survey there are:

1. polarised views surrounding interagency communication, with many not having formed an opinion around the design of SEND services and provision, and the ability of services to provide adequate support but that
2. professionals are confident in their own abilities to identify children with SEND, communicate with parents and signpost to services whilst
3. professionals report that they do not have enough time, nor resourcing, to be able to design and deliver good quality provision

In turn this perception corresponds to the main barriers which were identified within their roles. However, many felt that in contrast to perception and time allocation, training was sufficient. Professionals oddly rated their own abilities better than that of other professionals, which may reflect siloed working, poor inter‐agency communication or lack of time in which to be reflective. This may be due to self‐perception bias brought on as part of being part of a profession; all the years and investment in training would lead professionals to believe their job is better through self‐selection and perception, alongside any post‐cognitive dissonance with regards the choice. As part of SEND and care pathways is often fraught with emotivity it is also highly likely that professionals will be defensive and aim to inflate social opinions of their work. LAs were generally positive about service quality, which was at odds with all other professionals and is perhaps reflective of a more distant involvement.

Only slightly more than a third agreed or strongly agreed that inter‐agency communication around SEND was effective in their LA and reflects the conflicts and confusions within (and between) varying roles. Family members and participants repeatedly report poor interagency communication which leaves children without support (e.g., Farr et al. 2022). For example, LA professionals had the most positive view of overall communication whilst professionals within Education—the crux of the process—were the least positive. LA professionals are often removed from the day‐to‐day workings of provision, and so would not perhaps have the most realistic response as ever‐increasing austerity impacts those working more closely with children and their families (Kiely and Warnock 2023).

Sadly, high levels of confidence surrounding identification of SEND jar with reports of delays of up to 3.5 years within the last 10 years (Crane et al. 2016; Crane and Hill 2016), this may relate to formal assessment and documentation as opposed to the initial raising of concerns. As assessment and diagnosis are a core health profession contribution to the SEND process, it may explain their particularly high level of confidence. This is especially important, given the decline in the number of educational psychologists who are available to schools over the last decade (Atfield et al. 2023).

Strong agreement exists around barriers experienced across professions, as well as a marked social desirability bias within professions. Here, the findings suggest that professionals express their attitudes and frustrations such as the impact of training—whilst protecting their own professional space—which reflects prior work in the field (e.g., Cooper et al. 2016; Curran and Boddison 2021; Dobson 2023). Even so, this work builds on these pieces by showing how a resolution to the SEND crisis is going to be filled with complexity, with many similarities in the United Kingdom and abroad. As SEND is a particularly contentious and emotive subject, professionals are unsurprisingly guarded around views of their own profession, even when there is anonymity. This is a common occurrence where people wish to cultivate a positive image of themselves (Krumpal 2023; van de Mortel 2020). In the survey, there appears to be a gap between two views of professionals: the view of the *self as professional* and the view of others within *other services*. When rating others overall, professionals observe poor communication between professionals (62%), with insufficient time and resourcing (80%), leading to only half (50%) feeling services are designed effectively, with insufficient support provided (64%) as a result. However, when respondents referred to their own practice, all professions they were confident with identification (86%), communication to families (70%), signposting (80%) and with just about sufficient training (58%).

The discrepancy between professionals feeling they had sufficient training and insufficient resources is telling as it suggests professionals make do with what they have and believe the training given helps but are aware that this sits against the contradiction that training only can do so much—and that a barrier to better provision is due to lack of resourcing. For those professionals in particular who remember (in the Uinted Kingdom) pre and post‐the austerity era, this is particularly poignant. Funding models and outcomes (e.g., as reported in Male et al. 2020) show that a lack of funding is standard across most services, and yet the calculation for provision depends on whether teams are co‐located, how funding is calculated and how the make‐up of SEND as well as clinical teams is constructed. There is a significant difference in the cost, quality and speed with which systems work across the whole country as they work to achieve and increasingly neuro‐affirmative approaches. Professions appear to hold themselves in high regard whilst being negative towards other services that offer provision. This is in opposition to the 2014 reforms which were supposed to work against fractured service provision. There is a resultant need for staff to continue to be confident in their own skills whilst cultivating trust and positive multidisciplinary working relationships across service boundaries.

### 5.1 Methodological Issues

The co‐production of the survey questions ensures pertinence, but we recruited a convenience sample of respondents who chose to participate. Self‐reported data does have limitations; those with an interest in the topic are more likely to take part which can skew results away from a more balanced view and a representative population; it relies on individuals' ability to correctly recall information; social desirability can then skew further as a desire to appear in a certain way professionally may lead to inaccurate answers; this may introduce inconsistency in the way individuals interpret questions and introduce complexity across cultural and linguistic barriers and ignore deeply seated prejudices. Without a sample frame or knowledge of the response fraction, we cannot assess how representative or not our respondents might be of professionals working with children who have SEND. Respondents were also mostly female (87%), white (92%) and predominantly drawn from the Southeast and East of England (37%). In addition, these participants were predominantly drawn from the educational and health professions. Research shows that these numbers are commensurate with the occupations being surveyed; that is, vocational professions working in either health (66%) or education (76%) (Berlin et al. 2023; School Workforce in England, Reporting Year 2023 2023). Whilst our sample was moderately large, only 4% were from LAs, and there were insufficient numbers from social care to report their views as a single group, so their responses are included with ‘other professionals’. We did not formally assess the statistical differences so the differences reported between groups should be regarded as statistically significant.

### 5.2 Future Research

Future research needs to lean into the reasons why there appears to be continued division between and sometimes within professions that support children, parents, young people and families with SEND. Other than varying agencies (schools, health, LAs) which makes integrated working a challenge, often there are more practical reasons such as colocation of services which has been found to be useful within healthcare between child development teams (CDTs) and child adolescent mental health services (CAMHS) (Male et al. 2020). Further, there appears to be amongst professionals a protective layer of judgement for their own profession, and support that is given through training, whilst being critical of communication outside of their own profession, showing that communication barriers are the predominant reason for supporting the child which are often at odds, e.g., education vis a vis learning versus health. Social care professionals did not engage well with the survey, suggesting that whilst education and health are at least in dialogue, social care is still not fully integral to any conversations and collaboration. This leaves a key role in the integrated system without a voice. Families from minority backgrounds need to become a greater focus of representation. Longitudinal natural experiments are needed for SEND/health in future to isolate key factors to success which are currently unclear. Without consensus across these spaces, the resulting system leaves schools and health services to act unilaterally if any progress is to be made. For parents and children, the current framework to provision and support remains contorted and unclear. A pilot within a local area where health was placed within a school, or where models of activity and how these work within hospital schools could uncover many of the systemic issues that seem to plague SEND provision. Further, longer‐term stability in the allocation of provision, in a longer‐term pilot, one that stretches beyond a government's parliamentary term could avoid the constant sticking plasters of the last 25 years of review and reforms that seems to follow SEN provision.

## 6 Conclusion

Only a third of responding professionals agreed that interagency communication was effective, whilst most agreed that resourcing and time allocation lead to poorly designed services and insufficient support. Whilst the law has attempted to meet changing needs, it has been hogtied by reduced resourcing alongside increased demand. A decline in the number of Educational Psychologists in public service (Atfield et al. 2023), alongside changing expectations of the role of SENCOs (Curran and Boddison 2021; Rosen‐Webb 2011) and large‐scale reform in the NHS (NHS England 2019a, 2019b) all make for a perfect storm. Education professionals who work with children with SEND have in the last 30 years met wholesale SEND change at least four times or once every seven and a half years. Without role clarity and stability, alongside dwindling resources and increased demand, schools increasingly feel alone in offering provision, whilst LAs are increasingly distant, compounded by increasing academisation (Black et al. 2019). Whilst there are positive messages within the survey, these tend to refer back to the profession of the respondent whilst noting shortfalls with other services. A positive and truly integrated system needs to be created that cuts across the multidisciplinary nature of SEND. This system must be non‐hierarchical and responsive to need.

A whole systems change, e.g., Academisation redirects scant resources and files resourcing down cul‐de‐sacs for children with Special Needs as academies are not as accepting with adaptation of criteria for acceptance; this creates a more exclusive atmosphere which excludes children with SEND. Whilst training in theory should be better within a singular academy, this has not been the case as the destruction of singular LA has reduced the impact of change at scale as communities become divided as schools become run along business models. Meanwhile the NHS has yet again gone through another commissioning change, and funds are now taken from localised CCG to even larger Care Boards. So, whilst education has become fragmented through academisation, NHS funding (already fragmented through foundation trusts) is now trying to re‐unify resources in the opposite direction. This general sense of complex confusion leaves funding stranded and used wastefully on change dynamics.

Implications of this survey are fourfold: joint working and co‐location, integration of services across services so support is seamless, multi‐agency training (combining resource), systems sharing without blocks over sharing, local partnerships. Future revisions could be informed by seeing how long it takes for reform to reach adequate change, also by the use of effective data collection akin to the Autism data dashboard in the NHS being shared and implemented across education as well. Combining training across agencies with regard to particular needs (e.g., neurodevelopmental needs, ADHD and Autism).

Policymakers need to understand that professionals are strongly aware of resource‐scarce systems, and rationalisation creates instability in SEND provision, whereas stability must be a priority. Professional training and integration ultimately require a dedicated child health, education and social care agency for children with SEND; otherwise, it will be Groundhog Day once again (Thomas and Loxley 2022).

## Author Contributions

**William Farr:** conceptualization, data curation, investigation, formal analysis, writing – original draft, writing – review and editing. **Jennifer Saxton:** conceptualization, methodology, investigation, validation, formal analysis, supervision, visualisation, project administration, resources, writing – original draft, writing – review and editing. **Tamsin Ford:** conceptualization, methodology, data curation, investigation, validation, formal analysis, funding acquisition, writing – original draft, writing – review and editing. **Isaac Winterburn:** investigation, formal analysis, project administration, resources, writing – original draft, writing – review and editing. **Jacob Matthews:** investigation, formal analysis, project administration, resources, writing – original draft, writing – review and editing.

## Funding

This project is funded by the National Institute for Health Research (NIHR) under its ‘Programme Grants for Applied Research Programme’ (Grant Reference Number NIHR202025, The HOPE Study).

## Ethics Statement

Ethical approval was granted by the Cambridge Psychology Research Ethics Committee for this study (PRE.2021.058) in 2021.

## Conflicts of Interest

Tamsin Ford's research group receives funding for research methods consultation from Place2Be, a third sector organisation that provides mental health and training to UK schools. The other authors report no conflicts.

## Supporting information


**Appendix S1:** Supporting information.

**Appendix S2:** Supporting information.

**Appendix S3:** Supporting information.

**Appendix S4:** Supporting information.

**Data S1:** Supporting information.

**Data S2:** Supporting information.

**Data S3:** Supporting information.

## Data Availability

The data that support the findings of this study are available on request from the corresponding author. The data are not publicly available due to privacy or ethical restrictions.
