# Supplementary material for: Surveying the Professional Experience of Special Educational Needs Provision in England
Source: Child Care Health Dev. 2025 Dec 26;52(1):e70227. doi: 10.1111/cch.70227 (PMC12741706; doi:10.1111/cch.70227)
Supplement: Supplementary file 1 — Appendix S1: Supporting information. [file CCH-52-e70227-s004.docx]

**Participant Information Sheet for Stakeholder Online Focus groups**

Before you decide whether to take part in an online focus group discussion it is important for you to understand why they are being done and what your participation will involve. Please read the following information carefully and take time to decide whether you wish to take part. A member of the research team can also be contacted if you have any questions or if you would like more information. More details are at the end of this information sheet.

**Purpose of the study**

Children and young people with Special Educational Needs (SEN) often need extra support at school, college, and university. This is called ‘SEN Provision’. In order for SEN Provision to be put in place for young people, their special educational need(s) and requirement of additional support must first be identified. The extent of identified needs and the support that would most effectively meet them is then assessed by different professionals and/or specialists.

These three components make up the stages of the SEN process that our study is seeking to explore in more detail: 1. Identification, 2. Assessment, 3. Provision. We are collecting information from several different sources in relation to whether experiences of SEN provision are fair and equitable, and how experiences of these three stages make a difference to the health outcomes of children and young people. These focus groups are one of those sources.

**Why have I been chosen?**

We are inviting you to take part in an online focus group discussion because you took part in our online survey last year, and selected that you would like to be contacted about discussing your experiences related to the SEN process in more detail.

You therefore have knowledge and experience of working with families and young people who require SEN provision.

You will also fall into at least one of the following categories:

1. You are an education or health professional involved with the delivery of SEN provision in educational settings
2. You work for or closely with a Local Authority or Clinical Commissioning Group
3. You have worked in any of these professions within the last two years.

**Do I have to take part?**

No. You do not have to take part in the study. It is up to you to decide. If you decide to take part, you can withdraw from the study at any time without giving a reason. However, all information gathered up to that point will be kept, but not necessarily used unless you expressly ask us not to. No-one else will be informed if you choose to take part in the study.

**If I agree to take part, what happens next?**

After you have read this information letter, you will be asked to complete the consent form also attached to this email and send this back to the HOPE team via the following address: [hope21@medschl.cam.ac.uk](mailto:hope21@medschl.cam.ac.uk).

Once this document has been signed, please follow the URL link also included in this email to provide your current availability for attendance at different focus group discussions. You will be asked to provide your availability for as many sessions as possible. You will then receive an email in due course confirming the **one session** you have been allocated to attend based on your responses. If at any point, and for whatever reason, you are no longer able to attend this session, please inform us at the earliest convenience.

If we have not received your consent form, your provided availability will not be considered, and you will not be allocated to a session until this is received.

Based off previous communications about your areas of expertise, you will be invited to provide availability for attending a focus group about: IDENTIFICATION AND/OR ASSESSMENT AND/OR PROVISION AND/OR ALL.

Information about how to attend your allocated online focus group, including a Zoom invitation and house rules document will be provided in advance of the session.

Online focus group discussions will last **1.5 hours.**

Sessions will involve: an introduction from members of the research team and an ice breaker activity among participants; round-table discussions about several key topics within the allocated stage of the SEN process (Identification, Assessment, or Provision) where participants will share experiences in response to certain questions; a debrief and closing from members of the research team. These discussions will be audio recorded and anonymously transcribed by members of the research team.

There are no correct or incorrect answers and you will not be expected to answer every question. However, we would like to be inclusive of all opinions and hear from everyone at least once.

All participants will be reimbursed to the value of £40 in exchange for their time taking part in the study, either via a bank transfer or a Love2Shop voucher. A debrief form will be provided to participants with further information, including about sources of support.

At the end of the consent form there will be the option to provide an email address to be contacted if you would like to be kept informed about the study findings, or to hear about future opportunities for involvement. You do not have to provide your email address if you do not wish to, you will still be able to take part in a focus group discussion.

**Safeguarding Policy**

These conversations will be private and what you say will be kept confidential, unless the members of the research team you are speaking to think you are at risk of harm. In these instances, the team member will be legally obliged to report this to someone else, namely the University of Cambridge’s Designated Safeguarding Lead. However, this will not happen without your awareness and involvement. This process will be in accordance with standard operating procedures from the University of Cambridge.

**What are the possible benefits of taking part?**

If you decide to take part in the study, you will help us gather valuable information about the SEN process from the perspectives of professionals from across England. This information will be used to inform policy suggestions to improve SEN Identification, Assessment, and Provision for children and young people in the future.

**Are there possible disadvantages and/or risks in taking part?**

We do not think that there are any risks or disadvantages to taking part in an online focus group discussion.

You do not have to provide an answer to all of the questions if you do not want to provide comment. If you find answering questions about your experience of the respective SEN stage upsetting you can leave the call or asked to be moved into a breakout room for a short time, either verbally or via the chat function. Sources of support are listed at the end of this information sheet.

**Will my taking part in this project be kept confidential?**

After conclusion of the focus group session, all personal information will remain confidential, with actions in place to ensure that you cannot be identified in any data published by the HOPE study.

All focus group data, including a record of the meeting’s chatlog, will be collected and stored securely online through the University of Cambridge IT system. If you choose to provide your email address to be kept informed of the study’s findings or opportunities for future involvement, we will store your contact information separately from the focus group transcripts, and within the secure computer systems at the University of Cambridge. Only the immediate research team will have access to the focus group audio files, in which the team will assign unique IDs to all participants so you cannot be identified in any transcripts or publications.

You can find more information here about how the University uses personal data: <https://www.information-compliance.admin.cam.ac.uk/data-protection/research-participant-data>. You can also contact the research team for more information.

**What will happen to the results of the research project?**

We will be publishing the findings from the main HOPE Study in 2024. There are a few smaller studies within the HOPE study, such as these online focus group discussions, in which the results will also be published in 2024. We may publish our findings in journals, on the project website, on social media, and at conferences. However, your focus group data will be anonymised.

If you would like a copy of our findings, you will be able to get these from the project website (see below), or by email if you decide to provide your email address.

**Who is organising and funding the research?**

The HOPE Study is being carried out by the University of Cambridge and University College London, The University of Cambridge is in charge of the online focus group discussions. The HOPE Study is funded by the NIHR (National Institute for Health Research).

**Ethical** **review of the study**

The project has been reviewed by the University of Cambridge Psychology Research Ethics Committee **PRE:2021.058**.

**Contact for further information**

If you have any further questions or would like to take part in this project, please contact us or return your consent form at [hope21@medschl.cam.ac.uk](mailto:hope21@medschl.cam.ac.uk)

You can also find further details on our project website here: [HOPE Study: Health Outcomes for young People throughout Education - Child and Adolescent Resilience and Mental Health](https://dev.psychiatry.cam.ac.uk/hope-study-health-outcomes-for-young-people-throughout-education/)


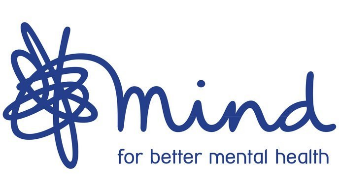
**Further support and information on mental health**


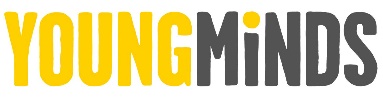


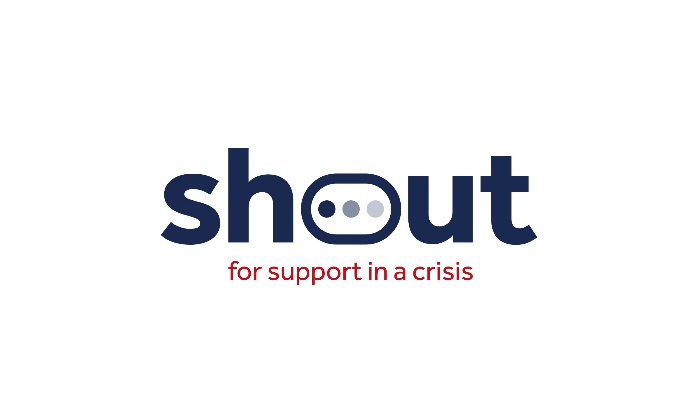

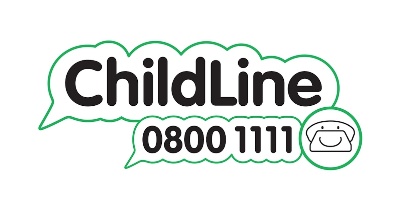
[https://www.min d.org.uk/](https://www.mind.org.uk/) <https://youngminds.org.uk/>

<https://www.giveusashout.org/> <https://www.childline.org.uk/>


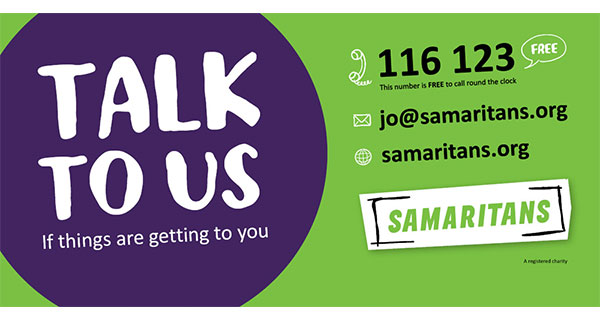


https://www.samaritans.org/how-we-can-help/contact-samaritan/
