# Supplementary material for: Surveying the Professional Experience of Special Educational Needs Provision in England
Source: Child Care Health Dev. 2025 Dec 26;52(1):e70227. doi: 10.1111/cch.70227 (PMC12741706; doi:10.1111/cch.70227)
Supplement: Supplementary file 2 — Appendix S2: Supporting information. [file CCH-52-e70227-s002.docx]

| **PLEASE HIGHLIGHT YOUR ANSWER TO EACH QUESTION** | | |
| --- | --- | --- |
| **Question** | **Response** | |
| 1. I confirm that I have read and understood the Participant Information Sheet | Yes | No |
| 1. I have had the opportunity to ask questions and had them answered   (If no, please contact the team at [hope21@medschl.cam.ac.uk](mailto:hope21@medschl.cam.ac.uk) and we will answer any of your questions) | Yes | No |
| 1. I understand that all personal information will remain confidential, with all efforts being made to ensure that I cannot be identified in any data.   (This means we will not share it with anyone outside of the research team) | Yes | No |
| 1. I understand that my data gathered in this study will be stored securely and kept confidential | Yes | No |
| 1. I agree that my data gathered in this study may be used for future research   (Selecting 'No' will not affect your participation in this focus group) | Yes | No |
| 1. I understand that my participation is voluntary | Yes | No |
| 1. I understand that I can withdraw my comments from the focus group at any point without giving a reason, up until and including the focus group itself (DATE TBC)   This can be done by contacting the research team at [hope21@medschl.cam.ac.uk](mailto:hope21@medschl.cam.ac.uk) | Yes | No |
| 1. I agree to take part in this focus group that focuses on the Identification of Special Educational Needs in children and young people | Yes | No |

**Consent Form - Identification Focus Group**

Participant signature

Please print your name

Date of signature / /

| **PLEASE HIGHLIGHT YOUR ANSWER TO EACH QUESTION** | | |
| --- | --- | --- |
| **Question** | **Response** | |
| 1. I confirm that I have read and understood the Participant Information Sheet | Yes | No |
| 1. I have had the opportunity to ask questions and had them answered   (If no, please contact the team at  [hope21@medschl.cam.ac.uk](mailto:hope21@medschl.cam.ac.uk) and we will answer any of your questions) | Yes | No |
| 1. I understand that all personal information will remain confidential, with all efforts being made to ensure that I cannot be identified in any data.   (This means we will not share it with anyone outside of the research team) | Yes | No |
| 1. I understand that my data gathered in this study will be stored securely and kept confidential | Yes | No |
| 1. I agree that my data gathered in this study may be used for future research   (Selecting 'No' will not affect your participation in this focus group) | Yes | No |
| 1. I understand that my participation is voluntary | Yes | No |
| 1. I understand that I can withdraw my comments from the focus group at any point without giving a reason up until (DATE TBC)   This can be done by contacting the research team at [hope21@medschl.cam.ac.uk](mailto:hope21@medschl.cam.ac.uk) | Yes | No |
| 1. I agree to take part in this focus group that focuses on the Assessment of Special Educational Needs in children and young people | Yes | No |

**Consent Form - Assessment Focus Group**

Participant signature

Please print your name

Date of signature / /

| **PLEASE HIGHLIGHT YOUR ANSWER TO EACH QUESTION** | | |
| --- | --- | --- |
| **Question** | **Response** | |
| 1. I confirm that I have read and understood the Participant Information Sheet | Yes | No |
| 1. I have had the opportunity to ask questions and had them answered   (If no, please contact the team at [hope21@medschl.cam.ac.uk](mailto:hope21@medschl.cam.ac.uk) and we will answer any of your questions) | Yes | No |
| 1. I understand that all personal information will remain confidential, with all efforts being made to ensure that I cannot be identified in any data.   (This means we will not share it with anyone outside of the research team) | Yes | No |
| 1. I understand that my data gathered in this study will be stored securely and kept confidential | Yes | No |
| 1. I agree that my data gathered in this study may be used for future research   (Selecting 'No' will not affect your participation in this focus group) | Yes | No |
| 1. I understand that my participation is voluntary | Yes | No |
| 1. I understand that I can withdraw my comments from the focus group at any point without giving a reason up until (DATE TBC)   This can be done by contacting the research team at [hope21@medschl.cam.ac.uk](mailto:hope21@medschl.cam.ac.uk) | Yes | No |
| 1. I agree to take part in this focus group that focuses on the Provision of Special Educational Needs support with children and young people | Yes | No |

**Consent Form - Provision Focus Group**

Participant signature

Please print your name

Date of signature / /
