# Supplementary material for: Surveying the Professional Experience of Special Educational Needs Provision in England
Source: Child Care Health Dev. 2025 Dec 26;52(1):e70227. doi: 10.1111/cch.70227 (PMC12741706; doi:10.1111/cch.70227)
Supplement: Supplementary file 3 — Appendix S3: Supporting information. [file CCH-52-e70227-s007.docx]

**Focus Group Topic Guide Draft 1: *Identification***

**Research Questions:**

1. **What are the effects of SEN provision on the Health Outcomes of Children and Young People with SEN?**
2. **Is SEND provision fair and equitable across England?**

**Instructions for facilitator**

- At the start of each session, go through the introduction, providing the background to the study, introductions to team members, and the house rules document
- There are X topics per session which are essential to cover, suggested timings are also included
- There are prompts and suggested follow-up questions under each topic. These are to be used at facilitators discretion.
- Following the close of the focus group discussion, there will be 30 minutes to reflect with team members and parent observer

**Introduction**

**Introduction from facilitator (5 minutes)**

- Reminder about recording and then *start recording*
- Introductions from team members in the call (Facilitator, Co-Facilitator, Parent observer)
- Background to study
- Introduce Focus Groups and the specific SEN Stage for discussion
- House rules
- Confidentiality and anonymity reminder (Don’t use names of CYP for instance)

**Participants introductions**

- Name
- Role
- Local Authority
- Background in I/A/P of SEN

**Ice breaker activity (5-10 minutes) choose one of:**

- **Future Headlines -** Ask each person to write a newspaper or magazine headline for the current state of SEN
- **Perfect dinner guest – choose one person from history that you’d love to have a meal with**
- **Bucket List -** Ask everyone to share their bucket list of things they want to do before they die.

**Identification Topic Guide**

**Core discussion points: (1 hour 10 minutes)**

1. In your current role please could you tell us how you are involved in the **Identification** of young people with SEN
2. Is there enough/adequate training for professionals for identifying SEN in your area?
   1. Is there anything missing/how could it be better?
   2. Are there enough resources to help professionals in this early stage?
3. How does communication between agencies impact on the process?
   1. How have you found working collaboratively with each of the agencies (education/health/LA)
   2. Is there anything missing/how could it be better?
   3. Are any changes required to the current system?
4. Does the age of identification have an impact on outcomes based on your experiences?
5. Which factors have the biggest influence on successful/best practice in Identifying young people with SEN in your LA
   1. How have you addressed these barriers in the past?
   2. What might need to change in the future?
6. Overall does the current system in your LA result in a more positive or negative outcome/experience for young people and their families?

**Further prompts:**

- Why do you think more parents are seeking a private diagnosis?
- Does the person (Parent/Teacher/Health Professional/ Young person) who first identifies that there may be a need for extra support have an impact on the process?
- In your LA what support is available to young people and their families in this part of the process?
- In your LA are young people and their families involved in this part of the process?
- If you could change one thing about Identification of SEN in your Local Authority, what would it be and why?

**Debrief (5 minutes)**

Thank participants for sharing their experiences with us and provide information for further communication with participants, study findings, reimbursement, reminders on confidentiality, who to contact with any further questions, complaints, or involvement in the project

**Post Focus Group Reflections (30 Mins)**

- Completion of reflections forms by facilitator and co-facilitator
- Feedback from parent observer and notes taken by co-facilitator

**Focus Group Topic Guide Draft 1: *Assessment***

**Research Questions:**

1. **What are the effects of SEN provision on the Health Outcomes of Children and Young People with SEN?**
2. **Is SEND provision fair and equitable across England?**

**Instructions for facilitator**

- At the start of each session, go through the introduction, providing the background to the study, introductions to team members, and the house rules document
- There are X topics per session which are essential to cover, suggested timings are also included
- There are prompts and suggested follow-up questions under each topic. These are to be used at facilitators discretion.
- Following the close of the focus group discussion, there will be 30 minutes to reflect with team members and parent observer

**Introduction**

**Introduction from facilitator (5 minutes)**

- Reminder about recording and then *start recording*
- Introductions from team members in the call (Facilitator, Co-Facilitator, Parent observer)
- Background to study
- Introduce Focus Groups and the specific SEN Stage for discussion
- House rules
- Confidentiality and anonymity reminder (Don’t use names of CYP for instance)

**Participants introductions**

- Name
- Role
- Local Authority
- Background in I/A/P of SEN

**Ice breaker activity (5-10 minutes) choose one of:**

- **Future Headlines -** Ask each person to write a newspaper or magazine headline for the current state of SEN
- **Perfect dinner guest – choose one person from history that you’d love to have a meal with**
- **Bucket List -** Ask everyone to share their bucket list of things they want to do before they die.

**Assessment Topic Guide**

**Core discussion points: (1 hour 10 minutes)**

1. In your current role please could you tell us how you are involved in the **Assessment** of young people with SEN
2. How does communication between agencies impact on the process?
   1. How have you found working collaboratively with each of the agencies (education/health/LA)
   2. Is there anything missing/how could it be better?
   3. Are any changes required to the current system?
3. Our surveys suggest that long waiting lists are the biggest barrier to best practice at this stage of the process, would you agree with this?
   1. What other barriers you have experienced?
   2. What would help to overcome these barriers?
4. Why do you think there has been an increase in the number of parents seeking a private assessment for their child?
   1. What is the impact of this?
   2. Does this make your job any easier?
5. Overall does the current system in your LA result in a more positive or negative outcome/experience for young people and their families?
   1. Why do you think over half of the parents who responded to our survey reported a very negative or negative experience during the Assessment stage?

**Further prompts:**

- In your LA is there enough support available to young people and their families in this part of the process?
- In your LA are young people and their families involved in this part of the process?
- Which aspects of the Assessment process make the biggest difference to children and young people, and why
- If you could change one thing about Assessment of SEN in your Local Authority, what would it be and why?

**Debrief (5 minutes)**

Thank participants for sharing their experiences with us and provide information for further communication with participants, Study findings, Reimbursement, Reminders on confidentiality, Who to contact with any further questions, complaints, or involvement in the project

**Post Focus Group Reflections (30 Mins)**

- Completion of reflections forms by facilitator and co-facilitator
- Feedback from parent observer and notes taken by co-facilitator

**Focus Group Topic Guide Draft 1: *Provision***

**Research Questions:**

1. **What are the effects of SEN provision on the Health Outcomes of Children and Young People with SEN?**
2. **Is SEND provision fair and equitable across England?**

**Instructions for facilitator**

- At the start of each session, go through the introduction, providing the background to the study, introductions to team members, and the house rules document
- There are X topics per session which are essential to cover, suggested timings are also included
- There are prompts and suggested follow-up questions under each topic. These are to be used at facilitators discretion.
- Following the close of the focus group discussion, there will be 30 minutes to reflect with team members and parent observer

**Introduction**

**Introduction from facilitator (5 minutes)**

- Reminder about recording and then *start recording*
- Introductions from team members in the call (Facilitator, Co-Facilitator, Parent observer)
- Background to study
- Introduce Focus Groups and the specific SEN Stage for discussion
- House rules
- Confidentiality and anonymity reminder (Don’t use names of CYP for instance)

**Participants introductions**

- Name
- Role
- Local Authority
- Background in I/A/P of SEN

**Ice breaker activity (5-10 minutes) choose one of:**

- **Future Headlines -** Ask each person to write a newspaper or magazine headline for the current state of SEN
- **Perfect dinner guest – choose one person from history that you’d love to have a meal with**
- **Bucket List -** Ask everyone to share their bucket list of things they want to do before they die.

**Provision Topic Guide**

**Core discussion points: (1 hour 10 minutes)**

1. In your current role please could you tell us how you are involved in the **Provision** of young people with SEN
2. In your experience do you think fellow professionals have enough training in providing support for young people with SEN?
   1. How could this be improved?
   2. What is missing?
   3. What are the barriers to training?
3. What do you think are the key services that need to be provided at this stage of the process?
   1. Are all of these provided in your LA? If not, why?
   2. What other services do you think would be beneficial?
4. In your experience does the provision as set out in young people’s EHCP’s match what they actually receive in school/education?
   1. Why do you think that is?
   2. What are the barriers to this?
   3. How could it be improved?
5. How does communication between agencies impact on the provision of support for young people with SEN?
   1. How have you found working collaboratively with each of the agencies (education/health/LA)
   2. Is there anything missing/how could it be better?
   3. Are any changes required to the current system?
6. Does your LA provide enough support services for young people and their families?
   1. Where do you signpost families to? Local Offer?
7. Overall does the current system in your LA result in a more positive or negative outcome/experience for young people and their families?

**Further prompts:**

- What do you find is the biggest difference in providing support for those young people with an EHCP compared to those who only have a SEN statement?
  - Has this changed over time?
- In your LA what support is available to young people and their families in this part of the process?
- In your LA are young people and their families involved in this part of the process?
- If you could change one thing about Provision of SEN in your Local Authority, what would it be and why?

**Debrief (5 minutes)**

Thank participants for sharing their experiences with us and provide information for further communication with participants, Study findings, Reimbursement, Reminders on confidentiality, Who to contact with any further questions, complaints, or involvement in the project

**Post Focus Group Reflections (30 Mins)**

- Completion of reflections forms by facilitator and co-facilitator
- Feedback from parent observer and notes taken by co-facilitator
