# Supplementary material for: Surveying the Professional Experience of Special Educational Needs Provision in England
Source: Child Care Health Dev. 2025 Dec 26;52(1):e70227. doi: 10.1111/cch.70227 (PMC12741706; doi:10.1111/cch.70227)
Supplement: Supplementary file 4 — Appendix S4: Supporting information. [file CCH-52-e70227-s001.docx]

**Parent/Carer Observer Notes**

| Notes from introduction: | | |
| --- | --- | --- |
| Examples of research question 1  **What are the effects of SEN provision on the Health Outcomes of Children and Young People with SEN?** | Examples of research question 2  **Is SEND provision fair and equitable across England?** | Other notable findings |
|  |  |  |

**Date of Focus Group:**

**Focus Group Topic: Identification/Assessment/Provision**
