# Supplementary material for: Surveying the Professional Experience of Special Educational Needs Provision in England
Source: Child Care Health Dev. 2025 Dec 26;52(1):e70227. doi: 10.1111/cch.70227 (PMC12741706; doi:10.1111/cch.70227)
Supplement: Supplementary file 5 — Data S1: Supporting information. [file CCH-52-e70227-s005.docx]

| Supplementary File 1. Questions for comparison between SEN Professionals | |  |
| --- | --- | --- |
|  | **Question** | **Response options** |
| Demographics | Gender | Male, Female, No gender, Non-binary, I prefer not to say |
|  | Region of England (Dropdown) | East Midlands, East Of England, London, North East, North West, South East, South West, West Midlands, Yorkshire And The Humber, I Don’t Know. |
|  | Ethnicity (Dropdown) | White English/ Northern Irish/ Scottish/ Welsh/British, White Irish, Irish Gypsy or Irish Traveller, Any other White background, Mixed/Multiple ethnic groups, White and Black Caribbean, White and Black African, White and Asian, Any other Mixed/Multiple ethnic background, Asian/Asian British, Indian, Pakistani, Bangladeshi, Chinese, Any other Asian background, Black/African/Caribbean/Black British, African, Caribbean, Any other Black/African/Caribbean background, Arab, Other ethnic group (please specify), I don't know, I prefer not to say |
|  | Main Occupation (Checkbox) | E.g Head Teacher, Special Educational Needs And/Or Disabilities Coordinator (SENCO/SENDCO), Teaching Assistant, Educational Psychologist, Occupational Therapist, Speech And Language Therapist, Paediatrician, General Practitioner (GP), Social Worker, Family Support Worker, Third Sector Or Charity Professional, |
| Noticing the need/Identification | In the LA/Practice where you spend most of your time, the agencies (health, social care, education) involved in SEND identification communicate effectively with each other about children and young people who may have SEND. (Likert Scale) | Strongly Agree, Agree, Neither Agree Nor Disagree, Disagree, Strong Disagree, N/A I Don’t Know, I Prefer Not To Say |
|  | I am confident in identifying children and young people with SEND (Likert Scale) | Strongly Agree, Agree, Neither Agree Nor Disagree, Disagree, Strongly Disagree, N/A Not Part Of My Remit, I Prefer Not To Say |
|  | I know where to signpost the parents and carers of children and young people when they are first identified as having SEND (e.g. to the Local Off er, or parent and carer forums) (Likert Scale) | Strongly Agree, Agree, Neither Agree Nor Disagree, Disagree, Strong Disagree, I Prefer Not To Say |
| Provision | What are the three main barriers to providing good quality SEND services, at the right time, for those who need them in the LA where you work(ed) most of the time? (Checkbox) | Choose three from the following 21 options:  Lack Of LA Funding, Lack Of Training Or Expertise, Lack Of Time, Lack Of Understanding Of The Processes Involved… |
|  | How do you feel the allocation of resources for SEND are influenced in the LA/Practice where you work(ed) most of the time? (Please tick one) (This includes those with and without an EHCP) (Checkbox) | Please tick one:  Based On First Come-First Served, By Need, By Severity, By LA Resources Available, On A Case-By-Case Basis, By Parent/Carer Ability To Advocate For Their Children, If A Child/Young Person Is In Care Or On The Edge Of Care, It Varies, Depending Who Is Assigned The Case, I'm Not Sure, I Prefer Not To Say |
|  | I am confident designing and providing services for all children and young people with SEND in the LA where I work(ed) most of the time (Likert Scale) | Strongly Agree, Agree, Neither Agree Nor Disagree, Disagree, Strongly Disagree, N/A Not Part Of My Remit, I Prefer Not To Say |
|  | I am confident in communicating about SEND provision with all the families of children and young people with SEND in the LA where I work(ed)most of the time (Likert Scale^4^) | Strongly Agree, Agree, Neither Agree Nor Disagree, Disagree, Strongly Disagree, N/A Not Part Of My Remit, I Prefer Not To Say |
|  | I am able to provide sufficient support to all the families of children and young people with SEND in in the LA where I work(ed) most of the time (Likert Scale) | Strongly Agree, Agree, Neither Agree Nor Disagree, Disagree, Strongly Disagree, N/A Not Part Of My Remit, I Prefer Not To Say |
|  | I have sufficient time and resources to design and deliver good quality SEND provision for all children and young people with SEND in the LA where I work(ed) most of the time (Likert Scale) | Strongly Agree, Agree, Neither Agree Nor Disagree, Disagree, Strongly Disagree, N/A Not Part Of My Remit, I Prefer Not To Say |
|  | I have sufficient training to design and deliver good quality SEND provision for all children and young people with SEND in in the LA where I work(ed) most of the time (Likert Scale) | Strongly Agree, Agree, Neither Agree Nor Disagree, Disagree, Strongly Disagree, N/A Not Part Of My Remit, I Prefer Not To Say |
