# Supplementary material for: Surveying the Professional Experience of Special Educational Needs Provision in England
Source: Child Care Health Dev. 2025 Dec 26;52(1):e70227. doi: 10.1111/cch.70227 (PMC12741706; doi:10.1111/cch.70227)
Supplement: Supplementary file 6 — Data S2: Supporting information. [file CCH-52-e70227-s003.docx]

**Supplementary File 2. Demographic characteristics of survey respondents (n=863)**

|  | | Group | | | | |
| --- | --- | --- | --- | --- | --- | --- |
|  |  | All SEN Professionals  N (%) | Education Professionals  N (%) | Health Professionals  N (%) | LA Professionals  N (%) | Other Professionals  N (%) |
| Gender | Male | 109 (12.6) | 95 (14.8) | 8 (6.7) | <5 | <5 |
|  | Female | 748 (86.7) | 542 (84.6) | 111 (92.5) | 63 (92.6) | 32 (94.1) |
|  | Other | 0 (0.0) | 0 (0.0) | 0 (0.0) | 0 (0.0) | 0 (0.0) |
|  | Prefer not to say | 6 (0.7) | <5 | <5 | 0 (0.0) | <5 |
| Region | East Midlands | 97 (11.2) | 76 (11.9) | 8 (6.7) | 11 (16.2) | <5 |
|  | East of England | 134 (15.5) | 90 (14) | 32 (26.7) | 8 (11.8) | <5 |
|  | London | 83 (9.6) | 56 (8.7) | 13 (10.8) | 10 (14.7) | <5 |
|  | North East | 50 (5.8) | 43 (6.7) | <5 | <5 | <5 |
|  | North West | 96 (11.1) | 78 (12.2) | 9 (7.5) | 6 (8.8) | <5 |
|  | South East | 187 (21.7) | 130 (20.3) | 32 (26.7) | 18 (26.5) | 7 (20.6) |
|  | South West | 77 (8.9) | 64 (10) | <5 | <5 | <5 |
|  | West Midlands | 81 (9.4) | 56 (8.7) | 13 (10.8) | <5 | 7 (20.6) |
|  | Yorkshire & the Humber | 56 (6.5) | 47 (7.3) | <5 | <5 | <5 |
|  | I don’t know | <5 | <5 | 0 (0.0) | <5 | 0 (0.0) |
| Ethnicity | White Ethnic Groups | 800 (92.7) | 597 (93.1) | 107 (89.2) | 65 (95.6) | 31 (91.2) |
|  | Mixed Ethnic Groups | 13 (1.5) | 9 (1.4) | <5 | 0 (0.0) | <5 |
|  | Asian Ethnic Groups | 24 (2.8) | 19 (3.0) | <5 | 0 (0.0) | 0 (0.0) |
|  | Black Ethnic Groups | 12 (1.4) | 8 (1.3) | 0 (0.0) | <5 | <5 |
|  | All other ethnic groups | <5 | <5 | <5 | 0 (0.0) | 0 (0.) |
|  | Prefer not to say | 10 (1.2) | <5 | <5 | 0 (0.0) | <5 |
