# Supplementary material for: Surveying the Professional Experience of Special Educational Needs Provision in England
Source: Child Care Health Dev. 2025 Dec 26;52(1):e70227. doi: 10.1111/cch.70227 (PMC12741706; doi:10.1111/cch.70227)
Supplement: Supplementary file 7 — Data S3: Supporting information. [file CCH-52-e70227-s006.docx]

**Supplementary File 3. Results from Questions 9 and 10**

|  | | Group | | | | |
| --- | --- | --- | --- | --- | --- | --- |
|  |  | All SEN Professionals  N (%) | Education Professionals  N (%) | Health Professionals  N (%) | LA Professionals  N (%) | Other Professionals  N (%) |
| Q9) What are the three main barriers to providing good quality SEND services, at the right time, for those who need them in the LA where you work(ed) most of the time? (Please tick three) | Lack of LA funding | 501 (19.9) | 400 (21.3) | 53 (14.8) | 30 (15.6) | 18 (18.6) |
|  | Lack of training or expertise | 131 (5.2) | 93 (5.0) | 21 (5.9) | 12 (6.3) | 5 (5.2) |
|  | Lack of time | 185 (7.3) | 131 (7.0) | 38 (10.6) | 12 (6.3) | 4 (4.1) |
|  | Lack of understanding of the processes involved | 53 (2.1) | 34 (1.8) | 10 (2.8) | 3 (1.6) | 6 (6.2) |
|  | Lack of information about the processes involved | 35 (1.4) | 30 (1.6) | 1 (0.3) | 3 (1.6) | 1 (1.0) |
|  | *Insufficient access to SEND specialists* | *351 (13.9)* | 286 (15.3) | 33 (9.2) | 24 (12.5) | 8 (8.2) |
|  | *Length of waiting lists* | *463 (18.4)* | 372 (19.9) | 49 (13.7) | 24 (12.5) | 18 (18.6) |
|  | Relationships with health professionals | 43 (1.7) | 33 (1.8) | 5 (1.4) | 5 (2.6) | 0 (0.0) |
|  | Relationships with LA professionals | 50 (2.0) | 42 (2.2) | 2 (0.6) | 3 (1.6) | 3 (3.1) |
|  | Relationships with social care professionals | 25 (1.0) | 17 (0.9) | 4 (1.1) | 3 (1.6) | 1 (1.0) |
|  | Relationships with education professionals | 12 (0.5) | 5 (0.3) | 5 (1.4) | 1 (0.5) | 1 (1.0) |
|  | Relationships with parents and carers | 20 (0.8) | 10 (0.5) | 4 (1.1) | 4 (2.1) | 2 (2.1) |
|  | Communication with parents and carers | 29 (1.2) | 12 (0.6) | 8 (2.2) | 6 (3.1) | 3 (3.1) |
|  | Communication between different services/intersectoral working | 168 (6.7) | 123 (6.6) | 25 (7.0) | 10 (5.2) | 10 (10.3) |
|  | Integration of services | 63 (2.5) | 37 (2.0) | 21 (5.9) | 5 (2.6) | 0 (0.0) |
|  | Excessive caseload | 251 (10.0) | 170 (9.1) | 48 (13.4) | 23 (12.0) | 10 (10.3) |
|  | Poor senior leadership | 29 (1.2) | 16 (0.9) | 6 (1.7) | 6 (3.1) | 1 (1.0) |
|  | Burnout | 30 (1.2) | 24 (1.3) | 4 (1.1) | 1 (0.5) | 1 (1.0) |
|  | Difficulties interpreting and applying the SEND Code of Practice | 24 (1.0) | 11 (0.6) | 5 (1.4) | 6 (3.1) | 2 (2.1) |
|  | Understanding of how to make reasonable adjustments | 50 (2.0) | 22 (1.2) | 15 (4.2) | 10 (5.2) | 3 (3.1) |
|  | None of these barriers apply in my LA | 7 (0.3) | 6 (0.3) | 0 (0.0) | 1 (0.5) | 0 (0.0) |
| Q10) How do you feel the allocation of resources for SEND are influenced in the LA/ Practice where you work(ed) most of the time? (Please tick one) (This includes those with and without an EHCP) | Based on first come-first served | 19 (2.20) | 15 (2.3) | 3 (2.5) | 0 (0.0) | 1 (2.9) |
|  | By need | 70 (8.1) | 49 (7.7) | 8 (6.7) | 10 (14.9) | 3 (8.8) |
|  | By severity | 48 (5.6) | 35 (5.5) | 10 (8.3) | 2 (3.0) | 1 (2.9) |
|  | By LA resources available | 205 (23.8) | 154 (24.1) | 26 (21.7) | 15 (22.4) | 10 (29.4) |
|  | On a case-by-case basis | 62 (7.2) | 47 (7.3) | 5 (4.2) | 8 (11.9) | 2 (5.9) |
|  | By parent/carer ability to advocate for their children | 239 (27.8) | 168 (26.3) | 43 (35.8) | 20 (29.9) | 8 (23.5) |
|  | If a child/young person is in care or on the edge of care | 9 1.0) | 8 (1.3) | 1 (0.8) | 0 (0.0) | 0 (0.0) |
|  | It varies, depending who is assigned the case | 97 (11.3) | 81 (12.7) | 6 (5.0) | 7 (10.4) | 3 (8.8) |
|  | I’m not sure | 106 (12.3) | 29 (12.3) | 18 (15.0) | 5 (7.5) | 4 (11.8) |
|  | I don’t know | 6 (0.7) | 4 (0.6) | 0 (0.0) | 0 (0.0) | 2 (5.9) |
